# Analysis of genetic structure and function of clustered regularly interspaced short palindromic repeats loci in 110 *Enterococcus* strains

**DOI:** 10.3389/fmicb.2023.1177841

**Published:** 2023-04-24

**Authors:** Shuan Tao, Dongdong Zhou, Huimin Chen, Na Li, Lin Zheng, Yewei Fang, Yao Xu, Qi Jiang, Wei Liang

**Affiliations:** ^1^School of Medicine, Jiangsu University, Zhenjiang, China; ^2^Department of Clinical Laboratory, Ningbo First Hospital, Ningbo, China; ^3^Department of General Medicine, Ningbo First Hospital, Ningbo, China; ^4^Bengbu Medical College, Bengbu, China; ^5^School of Medicine, Ningbo University, Ningbo, China; ^6^Department of Gastroenterology, Ningbo First Hospital, Ningbo, China

**Keywords:** gene structure, CRISPR-Cas system, drug resistance gene, *Enterococcus*, horizontal gene transfer (HGT)

## Abstract

Clustered regularly interspaced short palindromic repeats (CRISPR) and their CRISPR-associated proteins (Cas) are an adaptive immune system involved in specific defenses against the invasion of foreign mobile genetic elements, such as plasmids and phages. This study aims to analyze the gene structure and to explore the function of the CRISPR system in the *Enterococcus* genome, especially with regard to drug resistance. The whole genome information of 110 enterococci was downloaded from the NCBI database to analyze the distribution and the structure of the CRISPR-Cas system including the Cas gene, repeat sequences, and spacer sequence of the CRISPR-Cas system by bioinformatics methods, and to find drug resistance-related genes and analyze the relationship between them and the CRISPR-Cas system. Multilocus sequence typing (MLST) of enterococci was performed against the reference MLST database. Information on the drug resistance of *Enterococcus* was retrieved from the CARD database, and its relationship to the presence or absence of CRISPR was statistically analyzed. Among the 110 Enterococcus strains, 39 strains (35.45%) contained a complete CRISPR-Cas system, 87 CRISPR arrays were identified, and 62 strains contained Cas gene clusters. The CRISPR system in the *Enterococcus* genome was mainly type II-A (59.68%), followed by type II-C (33.87%). The phylogenetic analysis of the cas1 gene sequence was basically consistent with the typing of the CRISPR-Cas system. Of the 74 strains included in the study for MLST typing, only 19 (25.68%) were related to CRISPR-Cas typing, while the majority of the strains (74.32%) of MLST typing were associated with the untyped CRISPR system. Additionally, the CRISPR-Cas system may only be related to the carrying rate of some drug-resistant genes and the drug-resistant phenotype. In conclusion, the distribution of the enterococcus CRISPR-Cas system varies greatly among different species and the presence of CRISPR loci reduces the horizontal transfer of some drug resistance genes.

## 1. Introduction

Enterococci are Gram-positive facultative anaerobic cocci that constitute normal commensal microbiota of the human gastrointestinal tract (Attallah et al., 2022). In recent years, enterococci have become one of the leading causes of hospital-acquired infections and are common pathogens in various wound and surgical site infections (Farkas et al., 2022). The isolation rate of clinical strains Enterococcus is high among which *Enterococcus faecalis* (*E. faecalis*) and *Enterococcus faecium* (*E. faecium*) were the most prominent (Zhang et al., 2017). *Enterococcus* are intrinsically resistant to many first-line antimicrobial agents, such as clindamycin, cephalosporins, compound sulfonamide, and low-concentration aminoglycosides (Gołaś-Prądzyńska and Rola, 2021; Shahini Shams Abadi et al., 2021). In addition, *Enterococcus* can acquire drug resistance through gene horizontal genes transfer (HGT) mediated by mobile gene elements (MGEs) such as plasmids, phages, and transposons, making their resistance to macrolides, tetracyclines, quinolones, glycopeptides, and streptomycin increasingly common, which is one of the main mechanisms that contribute to the spread of resistance genes between bacteria (McInnes et al., 2020; Boccella et al., 2021). The ability of enterococci to acquire and transfer antibiotic resistance is a challenge in the clinical setting and makes it more difficult to treat nosocomial infections (Hegstad et al., 2010), especially since the even more alarming emergence of high mortality regarding vancomycin-resistant enterococci (Cattoir and Leclercq, 2013).

Clustered regularly interspaced short palindromic repeats (CRISPRs) are part of the adaptive immune system in diverse bacteria and archaea, which can recognize and cleave foreign DNA in a programmable and sequence-specific manner, and are disadvantageous for the HGT-driven spread of antibiotic resistance (Xu and Li, 2020; Wheatley and MacLean, 2021; Haider et al., 2022). CRISPR loci consist of short repeat sequences interspersed with unique spacer sequences that are homologous to sequences of invading DNA (“protospacers”) and a set of genes encoding nucleases (cas genes) are typically located near the CRISPR (Zhou et al., 2020). The leader sequence, located at the 5′ terminus of the CRISPR locus, is an AT-rich and relatively conserved sequence located upstream of the first repeat. The coordination of the components is fundamental to the normal function of CRISPR-Cas.

The CRISPR-Cas system is mainly divided into two classes and six types according to its constituent proteins and modes of action (Nishimasu and Nureki, 2017). Class 1 (type I, III, and IV) encode multi-subunit effector complexes, and Class 2 (type II, V, and VI) employ single protein effectors for target interference (Schwartz et al., 2022). The unique signature proteins of these six types are Cas3 for type I, Cas9 for type II, Cas10 for type III, Csf1 for type IV, Cpf1 for type V, and Cas13 for VI (Weissman et al., 2019). Genome analysis suggested that *E. faecalis* has a single type of CRISPR-Cas, type II (CRISPR1-Cas, CRISPR2, and CRISPR3-Cas) (Gholizadeh et al., 2021). The mechanism for type II CRISPR-Cas genome defense has been recently reviewed and is summarized as follows (Marraffini, 2015). Entry of exogenous DNA into the cell initiates the adaptation stage of type II CRISPR-Cas systems, a short fragment of DNA from the foreign element (protospacer) is acquired and incorporated as a novel spacer into the CRISPR array that functions as a genetic memory (de Freitas Almeida et al., 2022). During the expression stage, the CRISPR array is transcribed into a pre-CRISPR RNA (pre-crRNA) and processed into mature crRNAs by Cas9 assisted by RNaseIII and trans-activating RNA (tracrRNA) (Deltcheva et al., 2011). In the interference stage, Cas9 functions as a sole effector protein that binds to the crRNA/tracrRNA complex and cleaves target DNAs with a sequence complementarity to the protospacer (Shmakov S. et al., 2017). The matched proto-spacer and functional protospacer adjacent motifs (PAMs) (for discriminating self from foreign DNA) are necessary for CRISPR immune defense (Leenay et al., 2016).

The CRISPR-Cas system is a bacterial defense against foreign genetic elements, such as phages, plasmids, and transposons. Recent studies showed that the CRISPR-Cas system might play a role in antibiotic resistance in bacteria (Roy et al., 2021). Studies have shown that the CRISPR-Cas system in the *Streptococcus pneumoniae* was found to prevent plasmid transformation in the native state (Bikard et al., 2012). However, some researchers have found no evidence by statistical model that the CRISPR-Cas system can prevent the occurrence of HGT on the timescale of bacterial evolution (Gophna et al., 2015). Previous studies demonstrated type CRISPR-Cas systems both in limiting the acquisition of antibiotic resistance in *Klebsiella pneumoniae* (Li et al., 2018) and in enabling antibiotic resistance in *Francisella* (Sampson and Weiss, 2014). The effect of the CRISPR-Cas system on antibiotic resistance varies in different bacteria (Guo et al., 2022). Therefore, the study of the function of the CRISPR-Cas system and its effect on HGT and the relationship between CRISPR-Cas and antibiotic resistance should be more comprehensive and in-depth.

Bioinformatics analysis can help us understand CRISPR more fully. Systematic analysis of bacterial gene structure is of great significance for exploring more potential functions of bacteria. In recent years, with the continuous development of sequencing technology, the bacterial genome information included in GenBank is relatively large, which provides a rich source of data for our research, saves time and cost, and avoids a lot of repetitive sequencing work (Watanabe et al., 2018). In this study, we investigated the distribution of the CRISPR-Cas system in 110 *Enterococcus* isolates and multilocus sequence typing (MLST) and the drug resistance levels of selected strains. Furthermore, the relationships between CRISPR loci and multilocus sequence typing (MLST) and whether the CRISPR-Cas system plays a role in antibiotic resistance of *Enterococcus* were assessed to gain a deeper understanding of the system and its relationship with drug resistance.

## 2. Materials and methods

### 2.1. Source of *Enterococcus*

The whole-genome and gene annotation information of 110 *Enterococcus* strains were downloaded from the database of the National Center for Biotechnology Information (NCBI)^1^ and the CRISPR array was sourced using CRISPRFinder.^2^

### 2.2. Analysis methods

#### 2.2.1. Search and analysis of Cas genes

The search for the Cas gene in the *Enterococcus* genome was carried out using CRISPR-Cas Finder^3^, and the classification of the CRISPR-Cas system according to the type and composition of Cas gene contained in each strain was identified. The Cas gene locus was found by gene annotation of the whole genome sequence of *Enterococcus* downloaded from Nucleotide. Multiple sequences of the Cas gene sequence were performed using the ClustalW algorithm in MEGA11.0 software, which can be used to construct a phylogenetic tree.

#### 2.2.2. Analysis of sequences and RNA secondary structure of repeat sequences in CRISPR arrays

After the redundant direct repeat sequences (DRs) in the obtained CRISPR loci were removed, the DRs were aligned and analyzed by the ClustalW algorithm in MEGA11.0 and a phylogenetic tree was built. The RNA secondary structure of DRs and minimum free energy (MFE) of each group were predicted by the RNAfold web server^4^ with default arguments.

#### 2.2.3. Homology analysis of the spacer sequences

The number of spacers in CRISPR sites in each strain was counted. The targeted analysis of the spacer sequence of the CRISPR system was carried out by BLAST^5^ on NCBI. The targeting standard was set as the coverage and matching rates were both greater than 85%. The source and evolution of the spacer sequences were analyzed, and the relationship with the function and evolution of the CRISPR system was studied.

#### 2.2.4. Prediction and analysis of the leader sequences

Prediction and analysis of leader sequences were performed on structurally complete CRISPR systems containing the Cas gene. The sequence between the upstream of the first repeat sequence and the Cas gene was selected as the leader sequences search object, and the conserved region was found through ClustalW repeated comparison analysis. Conservation of leader sequences was predicted by the Weblogo web server.^6^ The possibility of promoter existence was predicted by Promoter 2.0.^7^

#### 2.2.5. MLST genotyping of *Enterococcus*

The MLST typing was found through the MLST database^8^, and the whole genome sequence collected in this study was submitted to the MLST database, which was divided into different sequence types (sequence type, STs) according to the seven housekeeping genes. The relationship between the ST typing and the CRISPR system type was analyzed.

#### 2.2.6. Screening of genome-wide drug resistance genes in *Enterococcus*

The prediction of resistance genes contained in the whole genome of *Enterococcus* infection was done by the RGI tool in the CARD database.^9^

### 2.3. Statistical analysis

Data analysis was done by SPSS 22.0 software. Whether the strains contain confirmed CRISPR arrays, Cas gene clusters, and complete CRISPR-Cas systems, as well as the relationship and difference between the carrier rate of drug resistance genes were tested by chi-square test and Fishers’ exact probability test; *p* < 0.05 was considered the difference to be statistically significant.

## 3. Results

### 3.1. Distribution of the CRISPR-Cas system in the *Enterococcus* genomes

In this study, a total of 110 *Enterococcus* genome sequences were collected. The distribution of the CRISPR-Cas system in the *Enterococcus* genomes is shown in Table 1. Among the collected strains, 59 (53.64%) strains had at least one CRISPR locus, 39 (35.45%) strains contained the complete CRISPR-Cas system (both CRISPR array and Cas gene cluster), and 20 strains had orphan CRISPR arrays (without Cas gene cluster). A total of 87 CRISPR loci were identified. The number of CRISPR loci found in different *Enterococcus* strains varied. Among the strains with CRISPR loci, the results showed that 37 strains of *Enterococcus* contained only one identified CRISPR locus, 19 strains contained two confirmed CRISPR arrays, and three strains each contained three, four, and five arrays. A single strain contained the highest number of CRISPR loci, which was five (*Enterococcus thailandicus a523*).

**Table 1:** Distribution of CRISPR-Cas system in 110 strains of *Enterococcus*.

Enterococcus	Number of strains	Confirmed CRISPR (strains)	Confirmed CRISPR strains (%)	Confirmed CRIPSR (number)	Cas positive (strains)	Cas positive strains (%)
*Enterococcus faecalis*	40	37	92.5	53	17	42.5
*Enterococcus faecium*	36	4	11.11	4	4	11.11
*Enterococcus avium*	2	1	50	1	1	50
*Enterococcus cecorum*	3	2	66.67	2	2	66.67
*Enterococcus durans*	6	2	33.33	2	6	100
*Enterococcus hirae*	10	7	70	12	10	100
*Enterococcus mundtii*	6	3	50	3	3	50
*Enterococcus silesiacus*	1	1	100	4	1	100
*Enterococcus thailandicus*	1	1	100	5	1	100
*Enterococcus* sp.	5	1	20	1	1	20
Total	110	59	53.64	87	46	41.82

There were 46 Cas cluster-positive strains. One strain (*Enterococcus faecalis CVM N60443F*) had two sets of different types of Cas genes (II-A + II-C). Other strains all contained one set of Cas genes. The CRISPR arrays and Cas gene clusters identified in *Enterococcus faecium* had lower carrier rates than other enterococci.

### 3.2. Distribution of Cas genes and the bioinformatics analysis of Cas1 gene sequences

In this study, a total of 46 of the 110 *Enterococcus* strains collected contained the Cas gene cluster, and the CRISPR-Cas system can be divided into four types, II-A, II-C, I-C, and I-B. The *Enterococcus faecalis CVM N60443F* genome contained a set of both II-A and II-C Cas genes. The remaining 45 strains contained only a cluster of Cas genes. A total of 47 sets of Cas genes were identified from 46 positive strains, of which 37 (80.43%) were II-A, six (10.87%) were II-C, two (4.35%) were I-C, and one (2.17%) was I-B.

**Figure 1:**
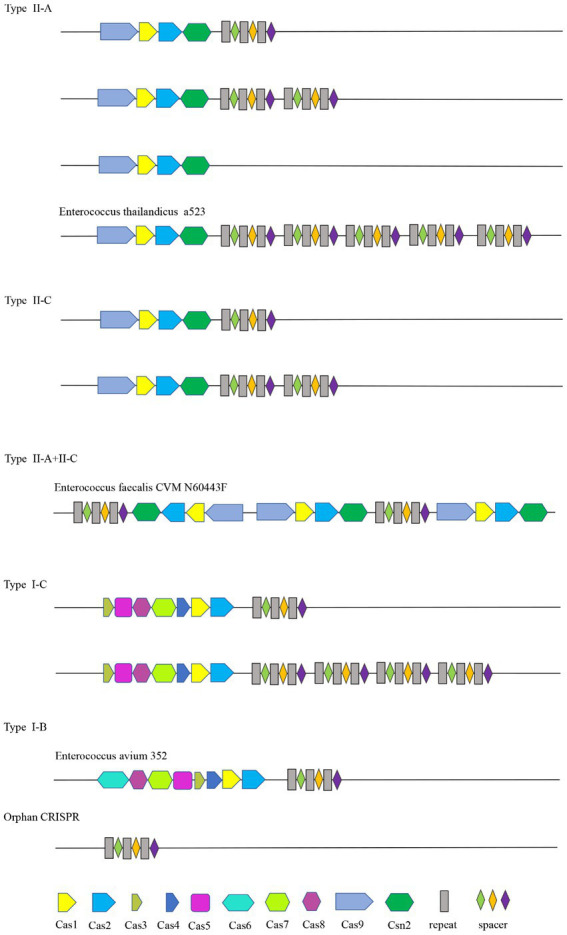
CRISPR structure distribution of *Enterococcus*.

The Cas gene clusters of the type II-A, II-C, I-B, and I-C CRISPR-Cas systems in *Enterococcus* were different. The Cas gene cluster of type I-B consists of Cas6, Cas8a, Cas7, Cas5, Cas3, Cas4, Cas1, and Cas2. Type I-C is Cas3, Cas5, Cas8c, Cas7, Cas4, Cas1, and Cas2. Cas1, cas2, csn2, and cas9 are shared by type II-C and type II-A CRISPR-Cas systems. Cas1 and cas2 are shared by four types. Strains containing the same type of CRISPR system had the same Cas gene clusters. The structure diagram of the CRISPR-Cas system in the *Enterococcus* genome is shown in Figure 1.

**Figure 2:**
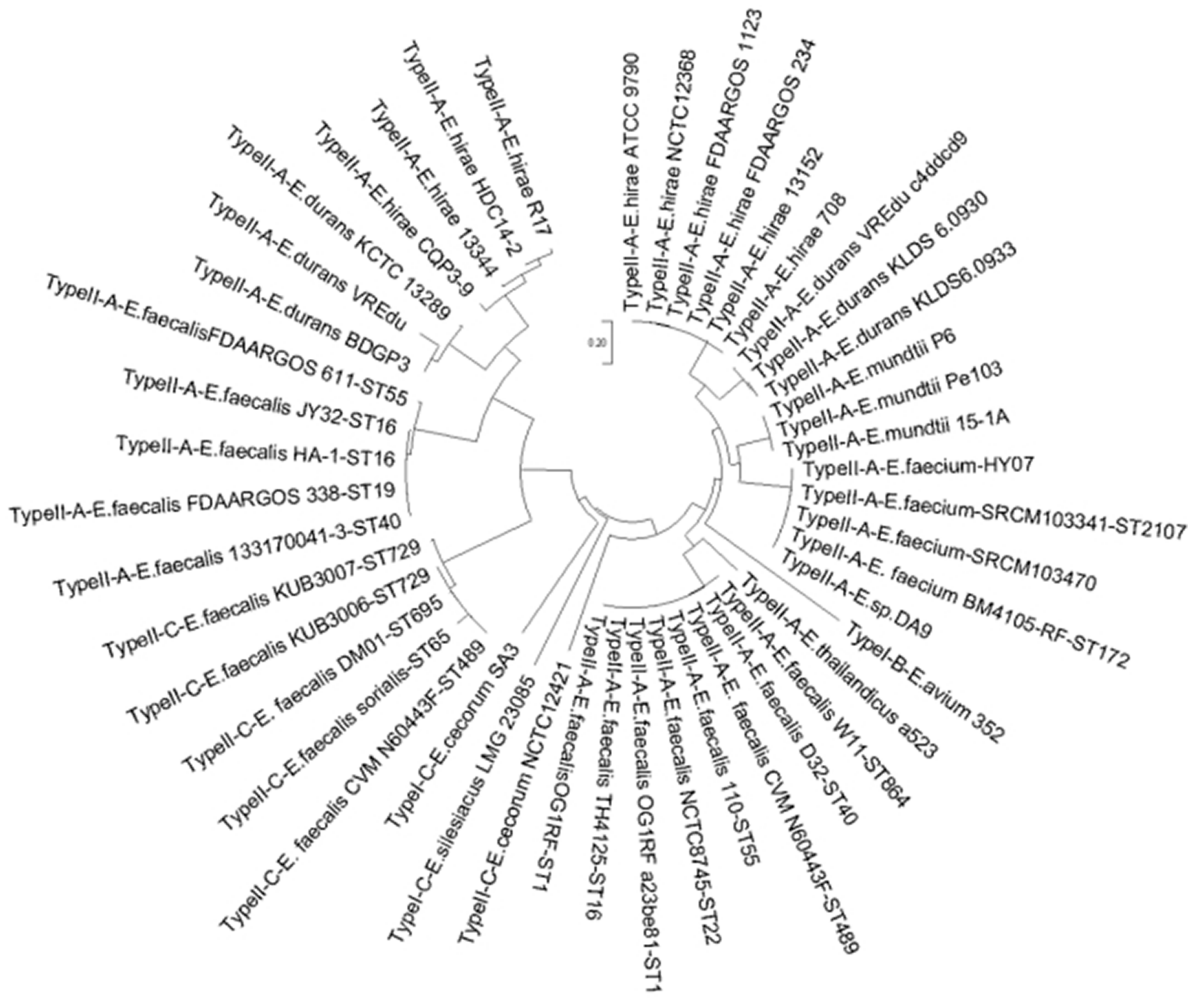
The phylogenetic tree based on *Enterococcus* 47 sets of cas1 genes. For the 47 sets of Cas 1 gene clusters contained in the genome of the 46 strains, the name format is CRISPR type + strain name + MLST type.

The acquisition of new spacers in the CRISPR system requires the involvement of the Cas1 protein. The length of the cas1 gene is about 865 bp and it is considered the core protein and relatively consistent. To further study the diversity and conservation of Cas gene sequences in *Enterococcus*, we aligned and analyzed the cas1 gene sequences and constructed a phylogenetic tree (Figure 2). The results showed that most bacteria existed on two branches, which was consistent with the typing results of the CRISPR-Cas system.

### 3.3. Analysis of sequences and RNA secondary structure of repeat in CRISPR arrays

#### 3.3.1. Analysis of repeat sequences in CRISPR arrays

**Figure 3:**
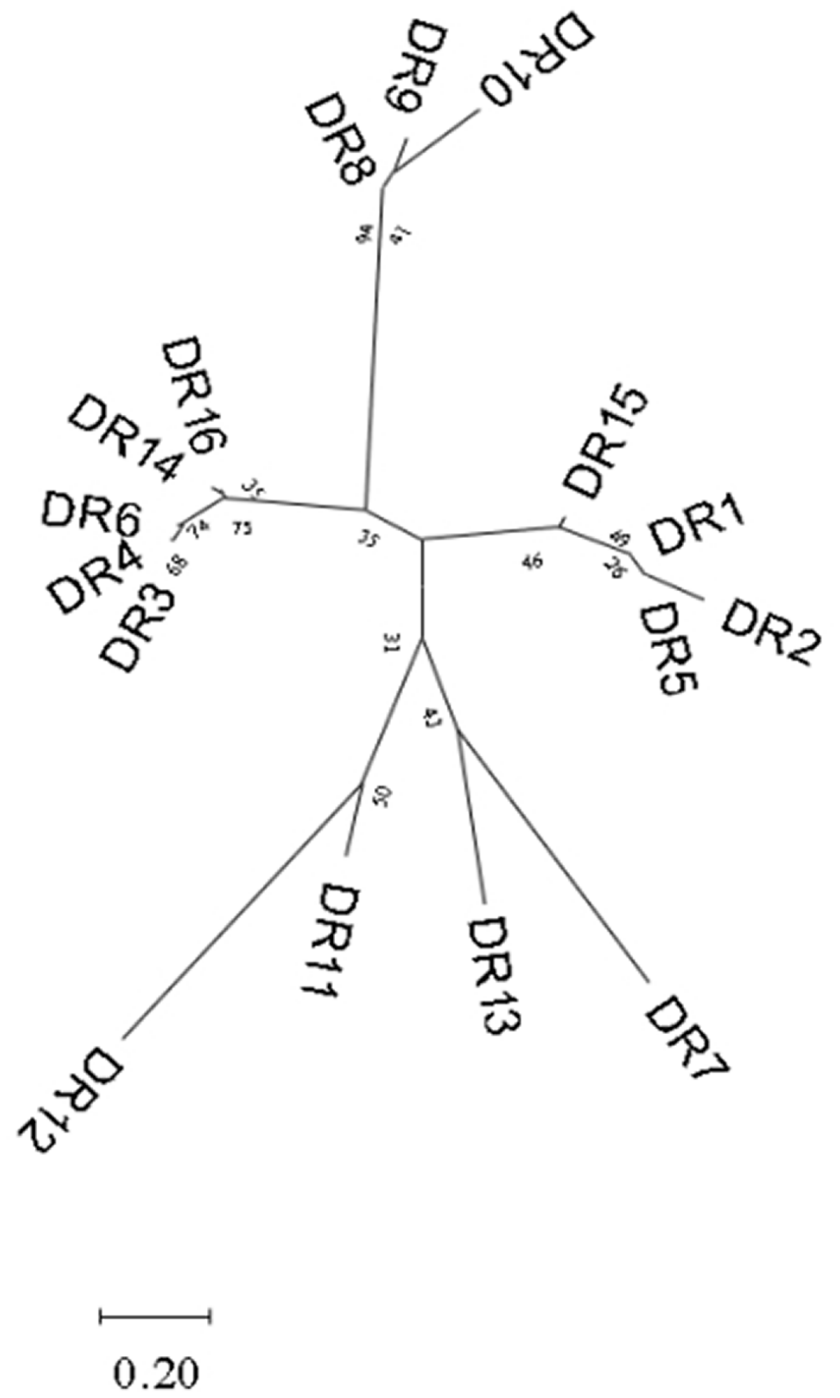
Phylogenetic tree constructed based on 16 groups of repeat sequences of *Enterococcus*. They can be divided into two groups.

A total of 87 different repeats were confirmed in 110 *Enterococcus* strains. The length of each direct repeat sequences (DRs) ranged from 26 to 39 bp, and the average length was 35.4 bp. The 87 repeat sequences can be divided into 16 groups by removing the redundant sequences, as shown in Table 2. DR1-7 belongs to type II-A, DR8-11 to type I-C, DR12-13 to type II-C, DR14 to type I-B, and DR15-16 cannot perform CRISPR system typing. Although the partial repeat sequences (DR5, DR7, DR16) appeared frequently in the CRISPR system of *Enterococcus*, it does not indicate that they are conserved among different *Enterococcus* species and different bacteria. The repeat sequences differed between various repeat types, but there was a certain degree of conservation within the same type, and there were differences in individual sequences or individual bases. In addition, we performed sequence alignment and phylogenetic analysis on the 16 repeat sequences and found that they can be roughly divided into two groups, one of which contains 14 repeat sequences, and the other which contains four repeat sequences (Figure 3).

**Table 2:** 16 groups repeat sequences of *Enterococcus* CRISPR array.

DR	Sequence	Length	*N*	Type
DR1	GGTTTTAGAGCTATGCTGATTTGAAT	26	1	II-A
DR2	CGTTTTAGAGCTATGTTGTTTTGAATG	27	1	II-A
DR3	CATTCAAAACAACATAGCTCTAAAAC	26	2	II-A
DR4	TTTTGGAAACATTCAAAACAACATAGCTCTAAAACC	36	1	II-A
DR5	GTTTTAGAGCTATGCTGTTTTGAATGCTTCCAAAAC	36	16	II-A
DR6	GTTTTGGAAGCATTCTAAACAACATAGCTCTAAAAC	36	6	II-A
DR7	GTTTTAGAGTCATGTTGTTTAGAATGGTACCAAAAC	36	13	II-A
DR8	GTCTCACCTTACATAGGTGAGTGGATTGAAAT	32	1	I-C
DR9	ATTTCAATCCACTCACTCTAGATTAGAGTGAGAC	34	1	I-C
DR10	ATTTCAATCCACTCACTCAATAAAGAGTGAGAC	33	1	I-C
DR11	ATTTCAATCCACTAGCTCTAAACAGAGCTAGAC	33	2	I-C
DR12	GTTTTTGTACTCTTAATGATGTGGTATCAGTAAAAC	36	1	II-C
DR13	GTTTTACTGATAAGAAATTATTGAGAGTACAAAAAC	36	5	II-C
DR14	GTCTAAATCTAAAAATAGTGGAATGTAAAT	30	1	I-B
DR15	TTGTTTTGGTACCATTCCAAACAACATGACTCTAAAACT	39	2	–
DR16	GTTTTGGTACCATTCTAAACAACATGACTCTAAAAC	36	33	–

#### 3.3.2. RNA secondary structure of repeat sequences

**Figure 4:**
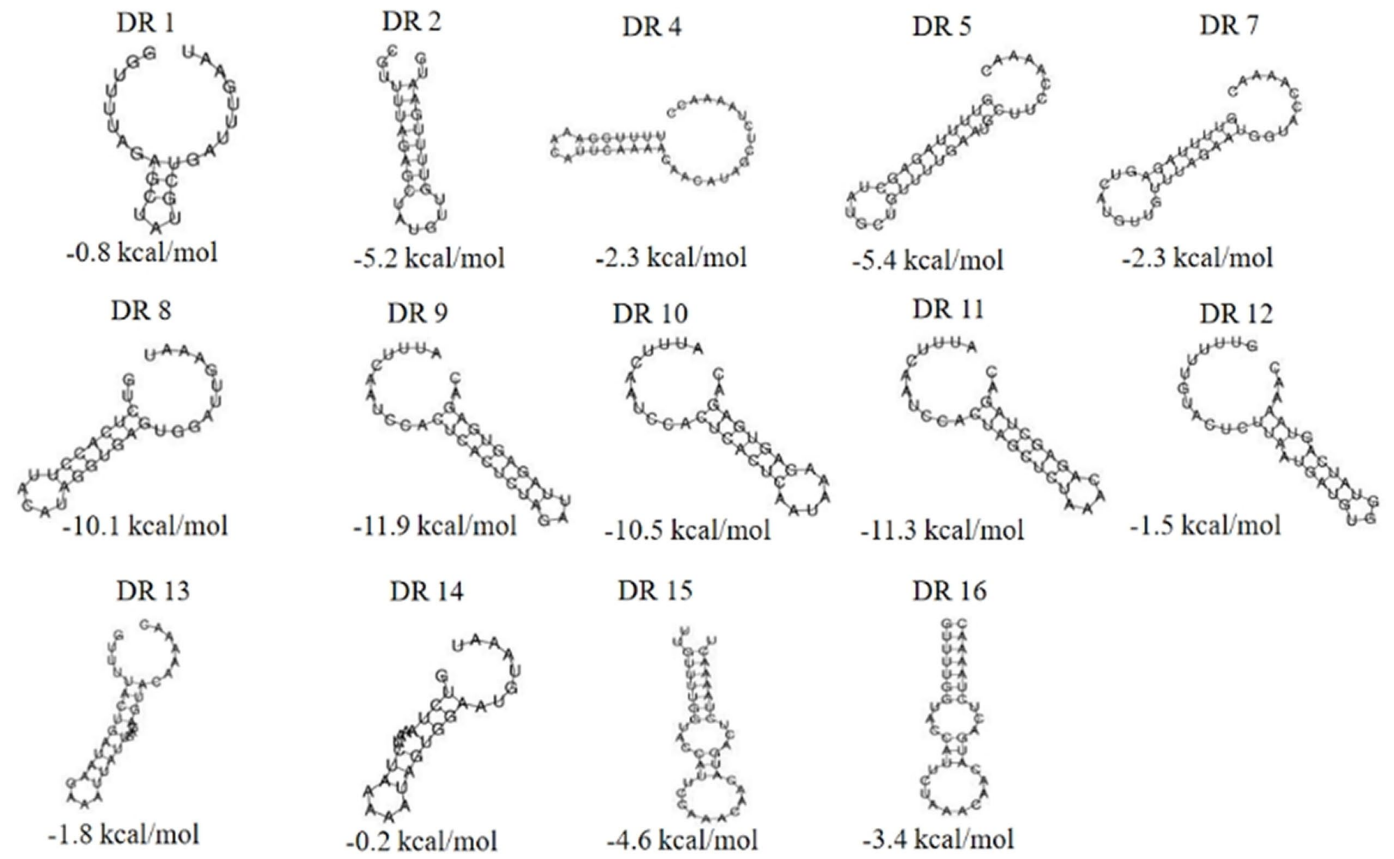
The RNA secondary structures and the MFE of the partial (14) repeats. Secondary structure prediction of the 16 repeat sequences by RNAfold and 14 repeat sequences can form structurally conserved dumbbell-shaped RNA secondary structures. The free energy of the thermodynamic ensemble was −0.8, −5.2, −2.3 -5.4, −2.3, −10.1, −11.9, −10.5, −11.3, −1.5, −1.8 −0.2, −4.6, −3.4 kcal/mol.

The RNA secondary structures and minimum free energy (MFE) of 16 groups of repeat sequences were predicted using the RNAfold web server (see Figure 4). It was found that there were 14 that can form structurally conserved dumbbell-shaped RNA secondary structures. The minimum free energy (MFE) value ranged from −11.9 kcal/mol to −0.2 kcal/mol. The DR12, DR13, DR14, and DR15 were complicated with three rings and two stems. DR16 had two rings at one end of the stem. The DRs in these groups were too long and the bases forming the stem were distributed at one end of the sequence. Other DRs contained a stem in the middle and a ring at each end. The lengths of the stems were 3–10 base pairs and the corresponding MFEs varied. The lower the MFE value, the more stable the structure. The stable secondary structure is beneficial to the immune function of the CRISPR system.

### 3.4. Homologous analysis of spacers

**Figure 5:**
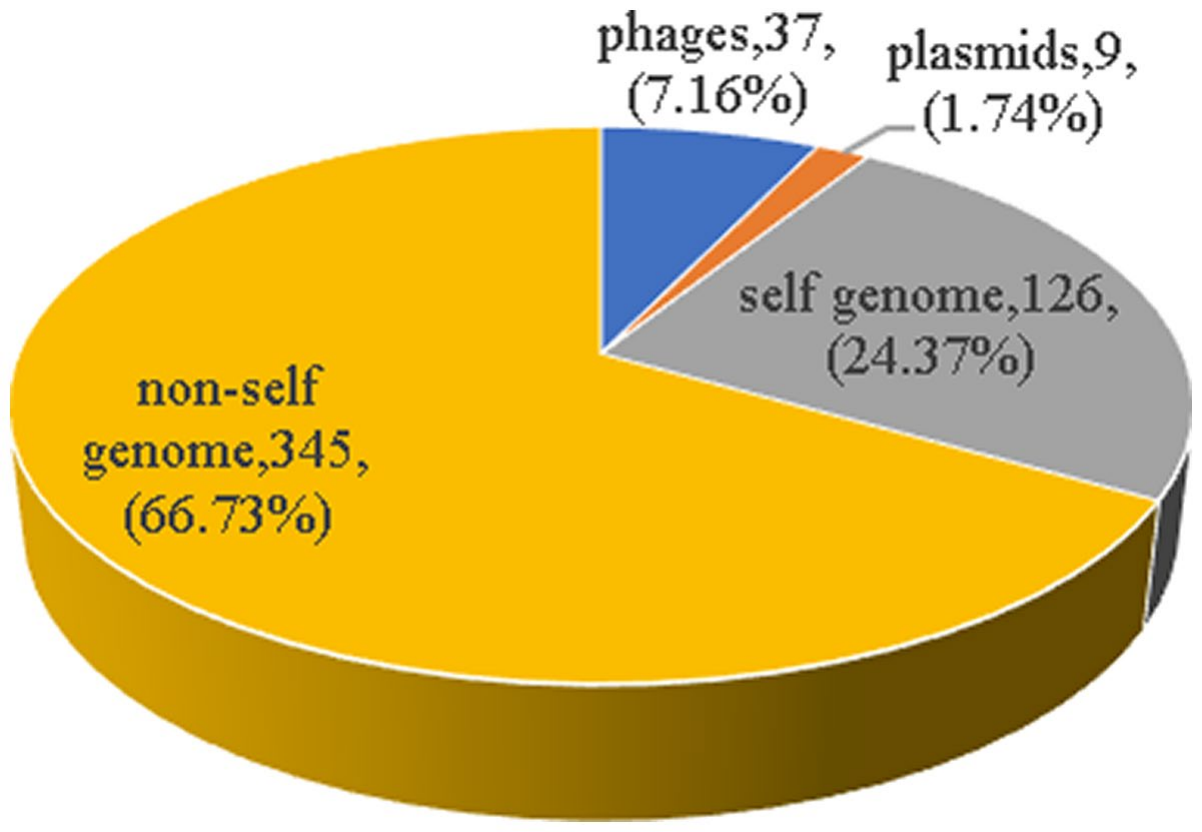
The homology analysis of spacer sequences. The homology of 517 spacer sequences in *Enterococcus* CRISPR was analyzed by BLAST alignment in GenBank. Nine (1.74%) were homologous to plasmids, 37 (7.16%) were homologous to phages, 126 (24.37%) targeted their own bacterial genomes, and 345 (66.73%) targeted non-self bacterial genomes.

There were a total of 622 spacer sequences in 110 strains of *Enterococcus* CRISPR array. Through multiple sequence alignment, 517 different spacer sequences were screened after removing the same sequence. The maximum number of these arrays was 50 spacer sequences, and the minimum was only one. The homology of 517 spacer sequences in *Enterococcus* CRISPR was analyzed by BLAST alignment in GenBank. The analysis found that nine (1.74%) were homologous to plasmids, 37 (7.16%) were homologous to phages, 126 (24.37%) targeted their own bacterial genomes, and 345 (66.73%) targeted non-self bacterial genomes (Figure 5). The majority (75%) of homologous plasmids were for *Enterococcus*, and 25% were for *Clostridium perfringens*. Homologous phages are basically *Enterococcus* phages. Some of the spacer sequences targeted a variety of phages simultaneously, such as the sequences in *Enterococcus faecalis* “CACTTTTAGCAGACTTGCTATAAAGCTTTT” which targeted 27 phages.

### 3.5. Prediction and analysis of the leading sequences

**Figure 6:**
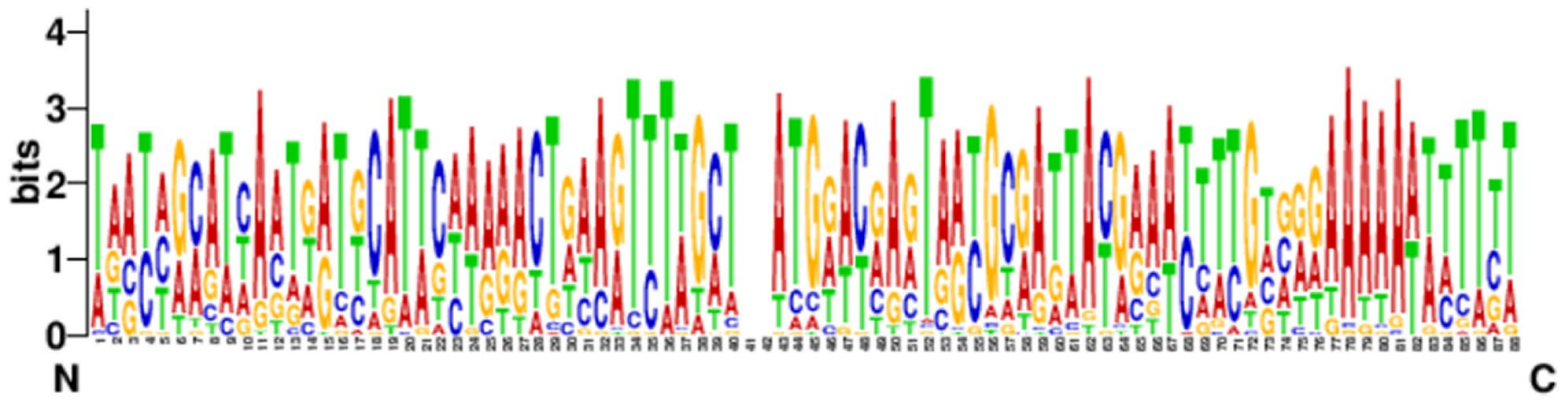
Analysis of the conservation of the leading sequences. The sequence identifier was created using WebLogo3, and the height of the letter indicates the relative frequency of the corresponding nucleotide at that position.

The leading sequences range in size from tens to hundreds of bp and are generally located upstream of the first repeat. The conservation of the leader sequence in 39 strains confirmed complete CRISPR structure which showed that the leader sequence was abundant in the AT bases with continuous “AAAA” and “TTTT” bases present, and with a palindromic structure. The leader sequence is highly conserved and the conserved region is located at the 3′ end, near the first repeat sequence in the locus (Figure 6). No promoter region was predicted by the Promoter 2.0 Prediction Server within the leader sequence.

### 3.6. The relationship between the CRISPR types and the MLST types

To understand the association between CRISPR-Cas types and STs among the selected strains, we retrieved MLST data from the MLST database. Due to the limitation of the database, we could only retrieve the MLST typing of *Enterococcus faecium* and *Enterococcus faecalis*, and the typing information of two strains of *Enterococcus faecalis* MLST was not available. These strains belonged to 56 different ST types, among which ST16, ST17, ST18, and ST117 covered a relatively large number—three, three, four, and three strains, respectively—and most STs covered a small number. The results observed that only 19 strains showed a relation with CRISPR typing and 55 strains of MLST typing were related to untyped CRISPR systems. The type II-A CRISPR system could be found in different *Enterococcus* MLST typing, among which ST16 type (21.43%) was the most common. II-C type was concentrated in ST65, 695, and 729 types, and ST729 (50%) was more abundant. Regardless of the distribution of the untyped CRISPR system, only the ST489 strain had both II-A and II-C types, and no cross-distribution was found in other types (see Table 3).

**Table 3:** The relationship between the CRISPR typing and MLST typing.

CRISPR typing	Number	MLST typing (Number)			
II-A	14	ST172(1)	ST2107(1)	ST864(1)	ST40(2)
		ST55(2)	ST16(3)	ST22(1)	ST1(2)
		ST19(1)			
II-C	4	ST65(1)	ST695(1)	ST729(2)	
II-A + II-C	1	ST489(1)			
Unclassifiable	55	ST22(1)	ST19(1)	ST17(3)	ST248(2)
		ST1137(1)	ST260(1)	ST209(1)	ST777(2)
		ST21(1)	ST179(1)	ST59(1)	ST64(1)
		ST74(1)	ST692(1)	ST97(1)	ST25(1)
		ST766(1)	ST191(1)	ST103(1)	ST9(1)
		ST30(1)	ST80(1)	ST18(4)	ST66(1)
		ST583(1)	ST1034(1)	ST904(1)	ST812(1)
		ST868(2)	ST54(1)	ST515(1)	ST976(1)
		ST32(1)	ST29(1)	ST6(1)	ST1165(1)
		ST330(1)	ST117(3)	ST673(1)	ST178(1)
		ST584(1)	ST121(1)	ST1141(1)	ST296(1)
		ST1985(1)			

### 3.7. The relationship between the CRISPR system and bacterial drug resistance

In this study, a total of 110 strains of *Enterococcus* were collected and the drug resistance genes carried in the genomes of each strain were retrieved, and their carrying rates in the confirmed CRISPR array, Cas gene, and complete CRISPR-Cas system were calculated. The relationship between the CRISPR system and the detection rates of bacterial resistance genes is shown in Table 4. The results showed that the carrying rate of the aminoglycoside resistance gene (*AAC(6′)-Ii*), efflux pump gene (*efmA*), the multidrug resistance gene (*eatAv*), and the vancomycin resistance gene (*VanC*) in the absence of the confirmed CRISPR arrays group (62.75, 41.18, 29.41, and 7.84%, respectively) were significantly higher than those containing confirmed CRISPR arrays group (10.17, 1.69, 8.47%, and 0, respectively). The carrying rate of the aminoglycoside resistance gene (*AAC(6′)-Ii*) and efflux pump gene (*efmA*) in the absence of the Cas genes group (50 and 32.81%, respectively) was significantly higher than the containing Cas genes group (13.04 and 2.18%, respectively). In addition, the carrying rate of the aminoglycoside resistance gene (*AAC(6′)-Ii*) and efflux pump gene (*efmA*) antibiotic resistance gene in the group without complete CRISPR-Cas system group (45.07 and 29.58%, respectively) was higher than that with the complete CRISPR-Cas system group (15.38 and 2.56%, respectively). The distribution differences were statistically significant (*p* < 0.05). The distribution of other drug-resistant genes was not statistically significant in the CRISPR-Cas group of this study.

**Table 4:** The relationship between the CRISPR system and bacterial drug resistance.

	CRISPR arrays			Cas genes			CRISPR-Cas		
Gene	Presence (*n* = 59)	Absence (*n* = 51)	*P*	Presence (*n* = 46)	Absence (*n* = 64)	*P*	Presence (*n* = 39)	Absence (*n* = 71)	*P*
AAC (6′)-Ii	6 (10.17%)	32 (62.75%)	0.000^*^	6 (13.04%)	32 (50%)	0.000^*^	6 (15.38%)	32 (45.07%)	0.001^*^
AAC (6′)-Iid	6 (10.17%)	3 (5.88%)	0.323	9 (19.57%)	0 (0)	0.000^*^	6 (15.38%)	3 (4.23%)	0.049^*^
AAC (6′)-Iih	2 (3.39%)	4 (7.84%)	0.413	6 (13.04%)	0 (0)	0.004^*^	2 (5.13%)	4 (5.63%)	0.640
dfrE	37 (62.71%)	3 (5.88%)	0.000^*^	17 (36.96%)	23 (35.94%)	0.535	17 (43.59%)	23 (32.39%)	0.168
dfrF	1 (1.69%)	3 (5.88%)	0.256	0 (0)	4 (6.25%)	0.110	0 (0)	4 (5.63%)	0.168
dfrG	3 (5.08%)	8 (15.69%)	0.063	3 (6.52%)	8 (12.5%)	0.243	3 (7.69%)	8 (11.27%)	0.406
efrA	38 (64.41)	3 (5.88%)	0.000^*^	18 (39.13%)	23 (35.94%)	0.443	18 (46.15%)	23 (32.39%)	0.111
efmA	1 (1.69%)	21 (41.18%)	0.000^*^	1 (2.18%)	21 (32.81%)	0.000^*^	1 (2.56%)	21 (29.58%)	0.000^*^
ErmB	8 (13.56%)	2 (3.92%)	0.075	7 (15.22%)	3 (4.69%)	0.060	7 (17.95%)	3 (4.23%)	0.022^*^
ErmT	0 (0)	2 (3.92%)	0.213	0 (0)	2 (3.13%)	0.336	0 (0)	2 (2.82%)	0.415
ErmG	1 (1.69%)	0 (0)	0.536	1 (2.18%)	0 (0)	0.418	1 (2.56%)	0 (0)	0.355
eatAv	5 (8.47%)	15 (29.41%)	0.004^*^	5 (10.87%)	15 (23.44%)	0.074	5 (12.82%)	15 (21.13%)	0.207
lnuG	1 (1.69%)	1 (1.96%)	0.715	1 (2.18%)	1 (1.56%)	0.664	1 (2.56%)	1 (1.41%)	0.585
lsaA	8 (13.56%)	0 (0)	0.005^*^	4 (8.70%)	4 (6.25%)	0.448	4 (10.26%)	4 (5.63%)	0.298
tetO	1 (1.69%)	1 (1.96%)	0.715	1 (2.18%)	1 (1.56%)	0.664	1 (2.56%)	1 (1.41%)	0.585
tetM	20 (33.90%)	4 (7.84%)	0.001^*^	10 (21.74%)	14 (21.88%)	0.588	10 (25.64%)	14 (19.72%)	0.313
tet(45)	2 (3.39%)	5 (9.80%)	0.163	3 (6.52%)	4 (6.25%)	0.624	2 (5.13%)	5 (7.04%)	0.520
tet(W/N/W)	1 (1.69%)	1 (1.96%)	0.715	1 (2.18%)	1 (1.56%)	0.664	1 (2.56%)	1 (1.41%)	0.585
vanA	1 (1.69%)	1 (1.96%)	1.715	2 (4.35%)	0 (0)	0.173	1 (2.56%)	1 (1.41%)	1.585
vanB	0 (0)	1 (1.96%)	0.464	0 (0)	1 (1.56%)	0.582	0 (0)	1 (1.41%)	0.645
vanC	0 (0)	4 (7.84%)	0.043^*^	0 (0)	4 (6.25%)	0.110	0 (0)	4 (5.63%)	0.168

*Represent statistically significant differences (*p*<0.05).

## 4. Discussion

The CRISPR-Cas system is an adaptive immune defense system evolved by bacteria and archaea to resist the invasion of foreign genetic material and is considered to be a natural obstacle to the spread of antibiotic resistance genes (Teng et al., 2021). The role of CRISPR-Cas in the spread of antibiotic resistance likely varies in different bacteria (Guo et al., 2022). In this study, we analyzed the gene structure of the *Enterococcus* CRISPR system and its potential function and explored its relationship with MLST and antibiotic resistance genes to further understand the function and drug resistance mechanism of the *Enterococcus* CRISPR system.

The complete CRISPR detection rate of *Enterococcus* reported in this study was 35.45%, which was lower than that of *Enterococcus* nosocomial isolates (46%) (Tao et al., 2022) and *Pseudomonas aeruginosa* (61.6%) (van Belkum et al., 2015), but higher than *Staphylococcus epidermidis* (7%) (Li et al., 2016). The presence of CRISPR-Cas in the *Enterococcus* genome was all lower than the average carrier rate of bacteria (45%) (Watson et al., 2021), which may be related to the loss of the CRISPR system in *Enterococcus* under antibiotic selection pressure. The complete CRISPR-Cas carrier rate of *E. faecium* (11.11%) was significantly lower than that of *E. faecalis* (42.5%). *Enterococcus faecalis* and *Enterococcus faecium* are the common clinical isolates of *Enterococcus*, and they are most closely related to the occurrence of diseases (Salzer, 2018). However, the content of the CRISPR-Cas system between the two is quite different, which is worthy of further study. An extensive comparative genomic analysis of *Enterococcus faecalis* and *Enterococcus faecium* revealed a direct association between the absence of the CRISPR-Cas system, the presence of endonuclease resistance (*ardA*), and the acquisition of vancomycin resistance in *Enterococcus faecium* (Mlaga et al., 2021). The presence of the CRISPR-Cas system is protective against the acquisition of specific mobile genetic elements carrying the vancomycin resistance genes (Jackson et al., 2017). However, the presence of the *ardA* gene inactivates the function of the endonuclease protective activity and makes the genome of *E. faecium* multifunctional in acquiring external DNA horizontally. Thus, it can regulate horizontal gene transfer, lead to multidrug resistance in *E. faecium* (Agudelo Higuita and Huycke, 2014; Palmer et al., 2014), and actively promote the acquisition and dissemination of antimicrobial resistance genes (Palmer et al., 2010). These observations may explain why *E. faecium* showed more resistance to vancomycin.

The Cas genes are generally located near the CRISPR loci and the encoded proteins play an important role in CRISPR adaptive immune function (Karah et al., 2015). In the type II CRISPR-Cas system, Cas9 is dispensable for acquisition and helps to select spacers with a correct PAM. The non-specific nuclease activity of Cas1 was the most conserved in nearly all CRISPR-Cas systems and required for adaptation, which can cleave the contiguous sequence, yielding a selected spacer sequence precisely. In our study, the proportion of *Enterococcus* Cas gene cluster positive strains was 41.48%. We also found some strains only have orphan CRISPR loci (no Cas gene) in the genomes. It has been suggested that orphan CRISPR arrays may be remnants of decaying CRISPR-Cas systems, or the CRISPR system causes the loss of Cas protein during the interaction with bacteria and foreign genes. The vast majority of orphan CRISPR are of unknown function (Price et al., 2016). Previous studies have pointed out MDR enterococci lack complete CRISPR systems (Palmer and Gilmore, 2010). Hullahalli et al. (2017) demonstrated that orphan CRISPR-Cas can provide genomic defense in the presence of functional CRISPR-Cas encoding factors.

Repeat sequences are mostly palindromic and highly conserved. The transcript of the repeats can form a hairpin structure and stabilize the overall secondary structure of the RNA. He et al. (2012) demonstrated that the stem-loop structure formed by the repeats may contribute to recognition-mediated contact between a gap-targeted exogenous RNA or DNA and a Cas-encoded protein, suggesting that the stability of RNA secondary structure may affect CRISPR function. It has been shown that CDRs from intact CRISPR-Cas sites are more likely to form stable RNA secondary structures with lower MFE compared to CDRs from an orphan CRISPR array (Wang L. et al., 2021). In our study, we found that the repeats are conserved in length and sequence, and the conservation is related to its classification and CRISPR-Cas system typing. However, similar results as described above were not found in the RNA secondary structure and MFE formed of repeat sequences from the complete CRISPR-Cas system and orphan CRISPR, which may be related to the strain information collected in this study. Studies have reported that repeat sequences can be used for bacterial typing (Shariat and Dudley, 2014; Toyomane et al., 2021) but, according to the cluster analysis results of repeat sequences in this study, we thought that the proportion of confirmed CRISPR arrays in *Enterococcus* and the number of repeat sequences is low, and the resolution not high, which are very limited if used for the typing of *Enterococcus*.

Spacer sequences serve as a memory bank for CRISPR-Cas systems to resist foreign nucleic acid invasion, and the number of spacer sequences within a locus can reflect the number of invasions to a certain extent (Shmakov S. A. et al., 2017). In this study, the minority spacers targeted plasmids and phage, indicating that *Enterococcus* has been less subjected to plasmids and phage invasion. The proportion of targeting exogenous genetic elements was lower than the reported in *Staphylococcus* (12.1%) (Zhang et al., 2019). The lower proportion of spacer sequences of *Enterococcus* matching with plasmids or phages in this study may be related to the limited number of plasmids or phages already sequenced and included in the Nucleotide database. Spacers in complete CRISPR-Cas systems exhibit high diversity in CRISPR loci compared to spacers in orphan CRISPR loci, consistent with the necessity of bacterial self-defense systems, partly demonstrating that the acquisition of new spacers is not immune to the existing effect of the interval (Wang Y. et al., 2021).

The leading sequences often vary in size, ranging from tens to hundreds of bp, mostly upstream of the 5′ end of the first repeat, usually a non-coding sequence consisting of contiguous AT structures that are identified in the same species (Alkhnbashi et al., 2021). It has also been suggested that the leader sequence may be the promoter for the transcription initiation of CRISPR arrays (Marraffini, 2015). Our study found that the *Enterococcus* leader sequence is relatively conservative and rich in AT bases, but the promoter was not predicted. The leader sequence in *Enterococcus* may have been mutated to cause the inactivation of the promoter, thereby inactivating the CRISPR-Cas system and allowing bacteria to acquire exogenous resistance genes to adapt to high antibiotic environments. It has been reported that the CRISPR system without a leading sequence is defective in transcription, but the specific cause of the deletion is not yet known (Marraffini, 2015).

MLST typing utilizes the nucleotide sequences of seven housekeeping genes of bacteria for typing and has gradually become a routine method for bacterial typing (Nutman and Marchaim, 2019).

In a study of the CRISPR-Cas system in *Klebsiella pneumonia*, the results indicated there was a significant MLST association with the distribution of the type I-E and I-E* CRISPR-Cas systems across *K. pneumoniae* (Li et al., 2018). Combined with our results, the connection between MLST typing and CRISPR requires further studies.

Enterococci are normal inhabitants of the human and animal gut but are listed as a global MDR pathogen by the World Health Organization with many antibiotic resistance traits located on plasmids and potentially spread by horizontal gene transfer (Arredondo-Alonso et al., 2020). Studies have shown that the CRISPR-Cas system has an important impact on the horizontal transfer of drug resistance genes (Serajian et al., 2021). The study by Palmer and Gilmore (2010) demonstrated that multidrug-resistant enterococci lack the CRISPR-Cas system, and the distribution of the CRISPR-Cas system varies greatly among different species. Clinical isolates of multidrug-resistant *E. faecalis* have more mobile genetic elements and lack the CRISPR-Cas defense system (MGEs) than commensal *E. faecalis* (Hullahalli et al., 2018). Previous studies on 16 *E. faecalis* draft genomes have indicated that there was a significant inverse correlation between the presence of CRISPR-Cas and acquired antibiotic resistance and examination of an additional eight *E. faecium* genomes yielded similar results for that species (Palmer and Gilmore, 2010). It was found that tetracycline resistance genes (*tetM, tet(45)*), macrolide antibiotic resistance genes (*ermA, ermB*) diamineopyrimidine antibiotic resistance genes (*dfrE, dfrF, dfrG*), and vancomycin resistance gene (*VanA, VanB, VanC*) etc. can be transmitted among enterococci bacteria and other bacteria through mobile genetic elements, thereby mediating the occurrence of drug resistance (Huang et al., 2021), which suggests that the CRISPR-Cas system has a non-negligible role in the spread of bacterial resistance genes. However, this study found that only the carriage of *AAC(6′)-Ii* and *efmA* resistance genes in enterococci were significantly higher in the lacking intact CRISPR-Cas group than in the intact CRISPR-Cas system, which may be related to the number of strains available for study with complete information being insufficient, or the strains selected according to the CRISPR database not being representative, which needs to be verified by further expansion of the sample size. Previous studies have confirmed that Cas proteins play an important role in the immune function of CRISPR-Cas (Gao et al., 2019), but whether the presence of Cas genes can affect the detection rate of bacterial drug resistance genes by affecting the adaptive immune function of the CRISPR system requires further research. Previous reports indicated the effect of CRISPR-Cas systems on antibiotic resistance varies in different species (Guo et al., 2022). In addition to the CRISPR system, the “innate immunity” of RM systems also plays a crucial role in immune defense (Makarova et al., 2013). A study on *E. faecalis* showed that CRISPR-Cas defense and RM defense have significant effects against plasmid genome defense (Price et al., 2016). Therefore, the role and mechanism of the CRISPR-Cas system in bacterial genomes and its role in bacterial populations and evolution still require more in-depth research.

This study analyzed the gene structure of the *Enterococcus* CRISPR-Cas system and explored the relationship with drug resistance genes in order to deeply understand the function and resistance mechanism of the *Enterococcus* CRISPR system. The results found in this research indicate that the presence of CRISPR loci can reduce the horizontal transfer of certain drug resistance genes, and the relatively low CRISPR system carrying rate in the genome may be one of the reasons for the increasing resistance rate of Enterococci and the greater susceptibility to multidrug resistance. Although the number of strains included in this study is limited, the analysis of the *Enterococcus* CRISPR system will help to understand its evolution direction, functional diversity, and typing analysis and lay the foundation for a more thorough exploration of the genetic mechanism of *Enterococcus* drug resistance and the relationship between CRISPR systems and antibiotic resistance.

## 5. Conclusion

In conclusion, this study mainly analyzed the structure of the CRISPR-Cas system and its relationship with MLST typing and drug resistance. The results found that except for *Enterococcus faecium*, the CRISPR-Cas system of *Enterococcus* has a higher carrying rate, revealed a certain degree of connection between the CRISPR-Cas system and MLST typing, and indicated that the presence of CRISPR-Cas is associated with the emergence of certain drug resistance genes and that CRISPR-Cas may hinder some drug resistance genes spread.

## Data availability statement

The original contributions presented in the study are included in the article/Supplementary material, further inquiries can be directed to the corresponding authors.

## Author contributions

ST: writing—original draft. QJ and WL: review and editing. HC, NL, YF, and YX: investigation. All authors contributed to the article and approved the submitted version.

## Funding

This study was supported by grants from the Natural Science Foundation of Jiangsu Province (BK20191210), the fifth phase of the “333 Project” scientific research project in Jiangsu Province (BRA2019248), the Jiangsu Commission of Health (H2018073), and the Subject of Lianyungang Science and Technology Bureau (SF2015).

## Conflict of interest

The authors declare that the research was conducted in the absence of any commercial or financial relationships that could be construed as a potential conflict of interest.

## Publisher’s note

All claims expressed in this article are solely those of the authors and do not necessarily represent those of their affiliated organizations, or those of the publisher, the editors and the reviewers. Any product that may be evaluated in this article, or claim that may be made by its manufacturer, is not guaranteed or endorsed by the publisher.

## Footnotes

^1^ http://www.ncbi.nlm.nih.gov/

^2^ https://crisprcas.i2bc.paris-saclay.fr/CrisprCasFinder/Index

^3^ https://crisprcas.i2bc.paris-saclay.fr/CrisprCasFinder/Index

^4^ http://rna.tbi.univie.ac.at/cgi-bin/RNAWebSuite/RNAfold.cgi

^5^ https://blast.ncbi.nlm.nih.gov/Blast.cgi

^6^ http://weblogo.berkeley.edu/logo.cgi

^7^ http://www.cbs.dtu.dk/services/Promoter/

^8^ https://pubmlst.org/

^9^ https://card.mcmaster.ca/
